# Development of 5-Substituted *N*-Methylmorphinan-6-ones as Potent Opioid Analgesics with Improved Side-Effect Profile

**DOI:** 10.1155/2012/208039

**Published:** 2012-06-17

**Authors:** Helmut Schmidhammer, Mariana Spetea

**Affiliations:** Department of Pharmaceutical Chemistry, Institute of Pharmacy and Center for Molecular Biosciences Innsbruck (CMBI), University of Innsbruck, Innrain 52a, 6020 Innsbruck, Austria

## Abstract

One of the most important functions of the opioid system is the control of pain. Among the three main opioid receptor classes (*μ*, *δ*, *κ*), the *μ* (MOR) is the main type targeted for pharmacotherapy of pain. Opioid analgesics such as morphine, oxycodone and fentanyl are agonists at the MOR and are the mainstay for the treatment of moderate-to-severe pain. However, adverse effects related to opioid use are severe and often lead to early discontinuation and inadequate analgesia. The development of more effective and safer medications for the management of pain still remains a major direction in pharmaceutical research. Chemical approaches towards the identification of novel MOR analgesics with reduced side effects include structural modifications of 14-alkoxy-*N*-methylmorphinan-6-ones in key positions that are important for binding, selectivity, potency, and efficacy at opioid receptors. This paper describes a representative strategy to improve the therapeutic usefulness of opioid analgesics from the morphinan class of drugs by targeting position 5. The focus is on chemical and biological studies and structure-activity relationships of this series of ligands. We report on 14-alkoxymorphinan-6-ones having a methyl and benzyl group at position 5 as strong opioid antinociceptive agents with reduced propensity to cause undesired effects compared to morphine although interacting selectively with MORs.

## 1. Introduction

**Figure 1:**
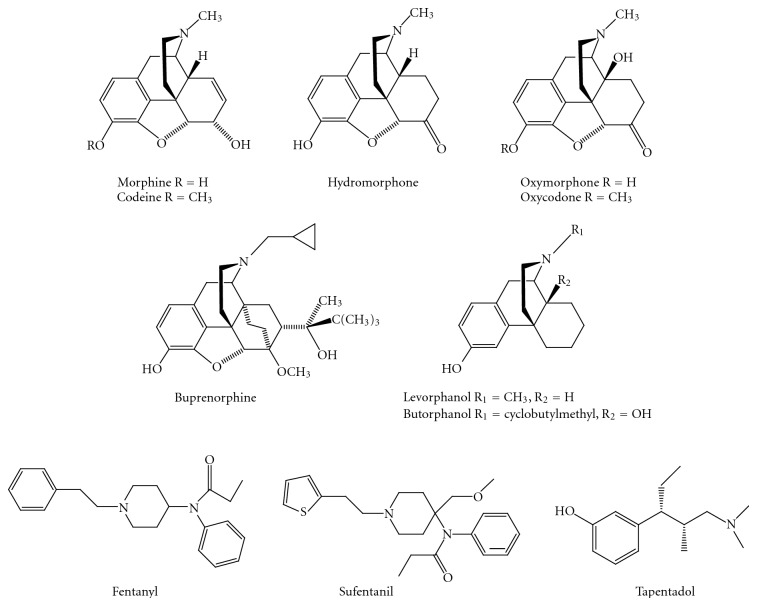
Examples of clinically used opioid analgesics.

The analgesic action of extracts of the opium poppy plant *Papaver somniferum* has been recognized for centuries. Morphine (Figure 1), the primary active component of opium, was isolated in 1805 by the German pharmacist Friedrich Sertürner, and more than 120 years elapsed when Gulland and Robinson proposed its correct structure [1]. Today, opioid analgesics play a central role in pain control and are generally considered to be highly effective in the management of moderate-to-severe pain [2, 3]. They can be classified into three classes: natural derivatives occurring in opium such as morphine and codeine; partially synthetic derivatives, including hydromorphone, oxycodone, oxymorphone, and buprenorphine; and synthetic compounds such as levorphanol, butorphanol, fentanyl, sufentanil, and the recently introduced tapentadol (Figure 1) [3–5]. 

Opioids act on three G-protein-coupled receptors (GPCRs) that is, *μ* (MOR), *δ* (DOR), and *κ* (KOR) [6], but it appears that the analgesic action of the most commonly used opioid analgesics is mediated primarily via the MOR. Activation of MORs, widely expressed in the nervous system and in peripheral tissues [7–10], is responsible not only for beneficial (analgesia) effects but also for a number of several undesired effects, which limits their clinical usefulness [3, 4, 11]. Adverse effects associated with opioid analgesics include respiratory depression, nausea, sedation, dizziness, vomiting, hypotension, and constipation. Long-term opioid use can cause tolerance, and thus complicating optimal pain treatment. Another concern with prolonged use of opioids is physical dependence and development of addictive disorders [4, 11]. Drug deaths from opioids are a serious and increasing issue [4]. On this basis, the development of more effective and safer medications for the management of pain, especially severe and chronic pain, still remains a major direction in pharmaceutical research.

**Figure 2:**
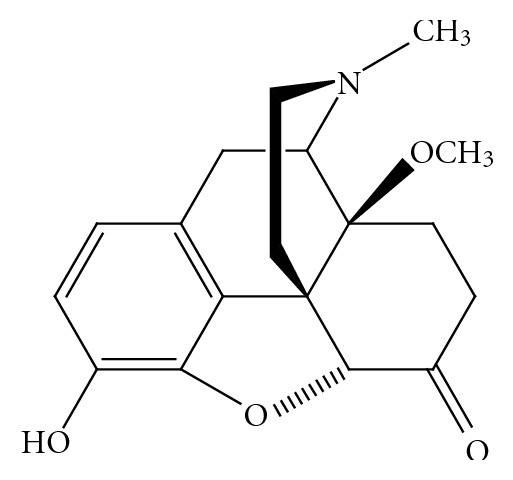
Structure of 14-*O*-methyloxymorphone **(1)**.

Chemical approaches towards the identification of novel MOR analgesics with reduced side effects are represented by structural modifications of morphinan-6-ones in key positions that are important for binding, selectivity, potency and efficacy at opioid receptors. A representative example is the development of the 14-*O*-methyl-substituted derivative of the clinically used MOR analgesic oxymorphone, namely, 14-*O*-methyloxymorphone (**1**) (Figure 2) [12]. It was reported that substitution of the hydroxyl group with a methoxy group in position 14 not only increases affinity to opioid receptors, while retaining the MOR selectivity of oxymorphone, but also markedly enhances the antinociceptive potency [12]. However, this MOR agonist induces the classical opioid unwanted effects of the conventional MOR analgesics [12–14]. An overview on synthesis and structure-activity relationships (SARs) on different 14-alkoxy-substituted morphinans has been recently published [15].

**Figure 3:**
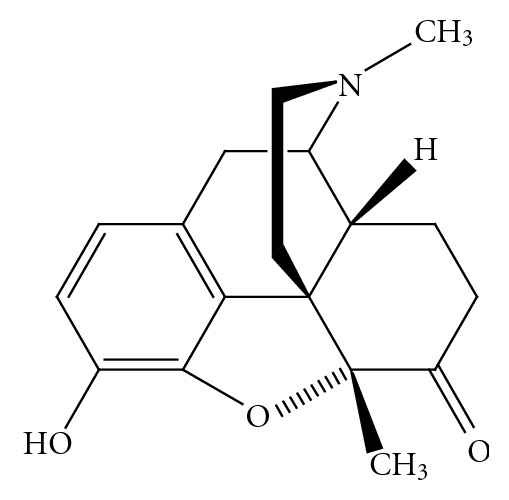
Structure of metopon.

An alternative strategy to improve the therapeutic usefulness of opioid analgesics from the morphinan class of drugs is to target position 5. The 5-methyl-substituted *N*-methylmorphinan-6-one, metopon (Figure 3), which shows higher analgesic potency than morphine, had also an improved side-effect profile concerning respiratory depression, physical dependence, mental dullness, tolerance, and nausea in patients [16, 17]. It was shortly used in an oral formulation for chronic pain relief in hospitalized cancer patients in the late 1940s [16]. Due to the low synthetical availability of metopon it was never introduced to the market as an analgesic drug. The positive preclinical and clinical findings on metopon represented a stimulating aspect for the design of differently substituted derivatives of metopon. Herein, we review recent advances on the development of 14-alkoxy substituted analogues of metopon, and also of a 5-benzylated analogue with agonist action at the MOR, as potent analgesics with reduced adverse effects. The focus is on SAR and pharmacological studies as well as on the description of synthetic procedures developed and used in the preparation of such derivatives.

## 2. Synthesis of 5-Substituted *N*-Methylmorphinan-6-ones

**Scheme 1:**
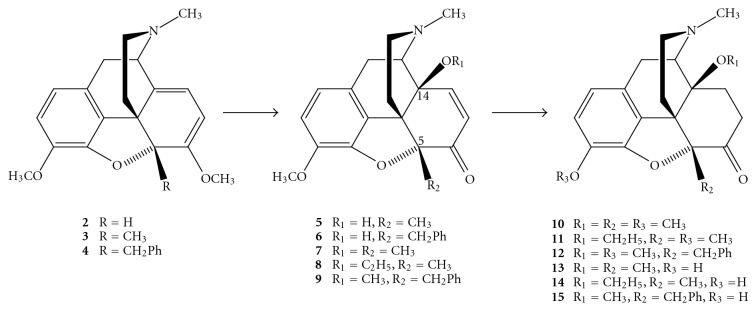
Preparation of 14-methoxymetopon (**13**), 14-ethoxymetopon (**14**), and 5-benzyl-14-*O*-methyloxymorphone (**15**).

 The starting point of extensive research in the area of *N*-methylmorphinan-6-ones with new substitution patterns was represented by the synthesis of 14-*O*-methyloxymorphone (**1**). While 14-*O*-methyloxymorphone (**1**) can be prepared starting from oxymorphone in four synthetic steps [12], 14-alkoxy-*N*-methylmorphinan-6-ones substituted in position 5 have to be prepared from thebaine (**2**) as starting material. Introduction of an alkyl substituent in position 5 of thebaine (**2**) can be accomplished by formation of the thebaine anion using *n*-butyllithium in THF at low temperature [18], followed by alkylation with the respective alkylating agent (methyl fluorosulfonate, dimethyl sulfate, or benzyl chloride), yielding 5-methylthebaine (**3**) and 5-benzylthebaine (**4**), respectively [18–21]. Treatment with performic acid afforded 14-hydroxy-5-methylcodeinone (**5**) [19, 20] and its 5-benzyl analogue **6** (Scheme 1) [14]. The *β*-orientation of the 14-hydoxy group was proved by X-ray analysis [20]. 14-O-Alkylation of **5** with dimethyl or diethyl sulfate in DMF in the presence of NaH gave the respective 14-alkoxycodeinones **7** and **8** which were catalytically hydrogenated to afford **10** and **11**. Ether cleavage of both compounds with 48% HBr solution yielded 77% of 14-methoxymetopon (**13**) and 75% of 14-ethoxymetopon (**14**) [19, 20]. Analogously, 5-benzyl-14-*O*-methyloxymorphone (**15**) (75% from **12**) was obtained from 14-hydroxy-5-benzylcodeinone (**6**) via intermediates **9** and **12** (Scheme 1) [14].

**Scheme 2:**
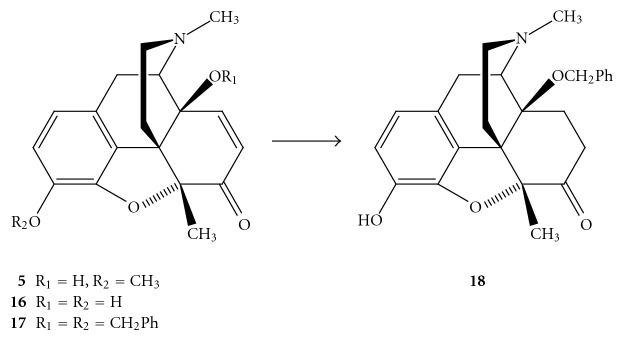
Preparation of 14-benzyloxymetopon (**18**).

**Scheme 3:**
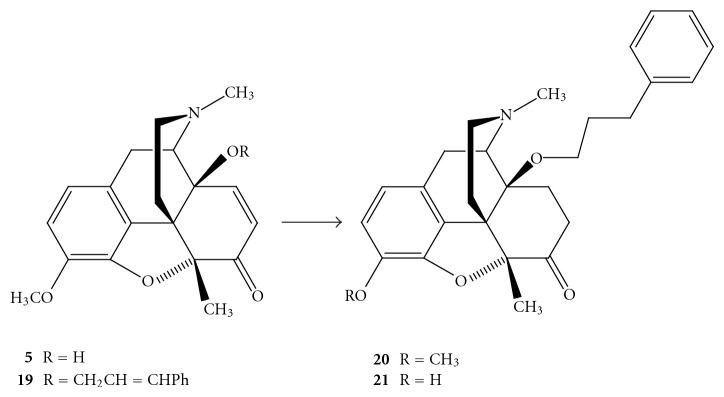
Preparation of 14-phenylpropoxymetopon (PPOM; **21**) and its 3-*O*-methyl ether (**20**).

14-Benzyloxymetopon (**18**) was prepared from 14-hydroxy-5-methylcodeinone (**5**). Compound **5** was first 3-O-demethylated using 48% HBr solution to give phenol **16** which was 3,14-bis-O-benzylated to afford compound **17**. Concomitant hydrogenation of the 7,8 double bond and hydrogenolysis of the 3-*O*-benzyl ether over Pd/C yielded 73% of **18** (Scheme 2) [13]. 14-Hydoxy-5-methylcodeinone (**5**) was also used for the synthesis of 14-phenylpropoxymetopon (PPOM; **21**) and its 3-*O*-methyl ether **20**. 14-O-Alkylation with cinnamyl bromide in DMF in the presence of NaH gave compound **19** which was catalytically hydrogenated to afford 73% of 3-O-methylated PPOM (**20**). Ether cleavage using 48% HBr solution yielded 88% of PPOM (**21**) (Scheme 3) [22].

## 3. Biological Activities of 5-Substituted *N*-Methylmorphinan-6-ones and Structure- Activity Relationship Studies

The applied strategy to obtain MOR analgesics in the *N*-methylmorphinan-6-one class exhibiting more favorable pharmacological features was initially based on the introduction of a small alkyl group such as methyl at the oxygen in position 14 of oxymorphone leading to 14-*O*-methyloxymorphone (**1**) [12]. This oxymorphone analogue displayed improved binding affinities at all three opioid receptors, while maintaining the MOR selectivity of the parent molecule, together with a considerable increase in in vitro and in vivo agonist potency (Table 1). In different in vitro functional assays including mouse vas deferens (MVD), guinea pig brain (GPI), and rat vas deferens (RVD) bioassays, and the [^35^S]GTP*γ*S binding assay using rat brain preparations, 14-*O*-methyloxymorphone proved to be a more potent MOR agonist than morphine and oxymorphone (Tables 1 and 2) [13, 14, 23, 24]. Compound **1** was also reported to possess up to 40 times higher antinociceptive potency than oxymorphone [12, 24], and it was up to 800 times more potent than morphine after subcutaneous (s.c.) administration in different pain tests in mice and rats (Tables 1 and 2) [12–14, 24]. Although it induced effective analgesia, this oxymorphone analogue **1** also produced the typical opioid adverse actions in mice after s.c. administration such as respiratory depression [12], physical dependence [12], inhibition of the gastrointestinal tract [13], and locomotor impairment [14].

Metopon (5-methyldihydromorphone; Figure 3) was reported to be about three times more potent than morphine as analgesic with lower tendency to produce nausea, sedation, respiratory depression, development of tolerance and dependence [16, 17]. Further chemical derivatization in the class of *N*-methylmorphinan-6-ones using 14-*O*-methyloxymorphone (**1**) as the lead, targeted position 5 by introducing a methyl group, giving rise to a new opioid compound, 14-methoxymetopon (**13**) [27]. 14-Methoxymetopon maintained the high affinity at the MOR in the subnanomolar range of its 5-unsubstituted analogue **1**, while DOR and KOR affinities were reduced by two to three times, resulting in higher MOR selectivity (Table 1). In in vitro bioassays, derivative **13 **was a potent agonist in the GPI (IC_50_ = 6.1 nM) [13], MVD (IC_50_ = 24.4 nM [13] and 12.7 nM [23]) and RVD preparations (IC_50_ = 268 nM) [24], showing comparable potency to 14-*O*-methyloxymorphone (**1**), and it was several times more active as agonist than morphine (Table 2). 14-Ethoxymetopon (**14**) was also described as MOR selective (Table 2), and a potent agonist in the GPI bioassay displaying similar potency to its 14-methoxy-substituted analogue **13**, and about 50 times greater than that of normorphine [25]. The potent MOR agonist activity of 14-methoxymetopon was also established using [^35^S]GTP*γ*S functional assays in rat brain [14, 26], calf striatum [28], and Chinese hamster ovary (CHO) cells expressing mouse MOR splice variants [28]. When compared to 14-*O*-methyloxymorphone (**1**), the 5-methyl analogue **13** showed less than three times lower agonist potency, while equal efficacy based on stimulation of [^35^S]GTP*γ*S binding in rat brain preparation (Table 2), signifying that methylation in position 5 did not considerably alter opioid agonist activity in vitro. 14-Methoxymetopon is available in tritium-labeled form ([^3^H]14-methoxymetopon) [26], which was described to have high affinity and selectivity for both native and recombinant MORs [26, 28]. In vivo agonist activities of 14-methoxymetopon (**13**) and its 14-*O*-ethyl analogue 14-ethoxymetopon (**14) **were also reported. Introduction of a methyl substituent in position 5 of 14-*O*-methyloxymorphone (**1**) resulted in a somewhat reduced analgesic potency in the mouse hot-plate and tail-flick tests after s.c. administration [13, 14, 24]. 14-Methoxymetopon and 14-ethoxymetopon were significantly more potent than morphine in producing antinociception (Table 1). A large number of studies reported on the analgesic properties of 14-methoxymetopon (**1**) in various pain models in mice, rats and dogs. These experiments were performed with different routes of administration, including s.c., intraperitoneal (i.p.), intravenous (i.v.), intracerebroventricular (i.c.v.), and intrathecal (i.t.), and employed a wide range of pain stimuli, that is, thermal, chemical, electrical and mechanical. The pain tests consisted of models of acute nociceptive pain such as hot-plate [13, 14, 25, 29, 30], tail-flick [14, 25, 30–32], tail-electrical stimulation [33], and skin-twitch [34], visceral pain (acetic acid- [25, 27] and paraphenylquinone (PPQ-) induced writhing [30]), and inflammatory pain (carrageenan-induced inflammatory hyperalgesia [35]). 14-Methoxymetopon induced effective analgesia in all models of pain in rodents, showing different degrees of higher potency than morphine, depending on the analgesic paradigm and route of administration used (Table 3). In dogs, it had similar antinociceptive efficacy to sufentanil in the skin-twitch test after i.v. application [33]. 14-Methoxymetopon (**13**) significantly reduced pain-behavior in response to heat and mechanical stimulation in the inflamed paw of rats with carrageenan-induced inflammatory hyperalgesia [35].

14-Methoxymetopon (**13**) was generally described to produce less severe adverse effects than traditional MOR analgesics. The gastrointestinal inhibitory activity of this derivative after s.c. administration was relatively weak, its maximal inhibition was only approximately 65%, whereas morphine completely blocked the transit in the charcoal test in mice [31]. Intravenous administration of 14-methoxymetopon (**13**) does not induce respiratory depression compared to sufentanil when given in equianalgesic doses to dogs [34]. A dose of 12 *μ*g/kg sufentanil decreases oxygen and carbon dioxide tension (PaO_2_ and PaCO_2_) by 41% and 57%, respectively, while at the same dose of 14-methoxymetopon, gas levels remain almost unchanged [34]. In the same study using conscious dogs, it was shown that the MOR opioid agonist **13** evokes significantly reduced bradycardia and hypotension, and produces less sedation than sufentanil [34]. The maximal bradycardic effect was 19% after 14-methoxymetopon and 42% after sufentanil, and the maximal decrease in the mean arterial blood pressure was 20% after sufentanil, and only 6% after derivative **13** at the highest i.v. dose in dogs [34]. The physical dependence liability of 14-methoxymetopon in the naloxone-induced withdrawal jumping test in mice was much lower than that of morphine when administered i.p. in equianalgesic doses with 78% response showed by morphine-treated animals and 38% by mice receiving derivative **13** [25]. Similar observations on the induction of minimal physical dependence compared to morphine were made in rats not only for 14-methoxymetopon (**13**) but also for its 14-ethoxy substituted analogue, 14-ethoxymetopon (**14**) in naloxone-induced abstinence, both MOR agonists show negligible withdrawal syndromes [25]. The rate of development of tolerance to analgesia was markedly slower than that of morphine. In the tail-flick test in rats, a much lower degree of tolerance was reported after chronic treatment with compound **13** for 7 and 11 days than morphine when given i.p. in equianalgesic doses [25, 32]. Also, 14-ethoxymetopon (**14**) failed to induce significant tolerance to the antinociceptive action [25]. Additional behavioral studies described that derivative **13** is more effective in rats in reducing the emotive/affective component of pain and in producing an anxiolytic effect than morphine in the elevated-plus maze [33]. Despite 14-methoxymetopon's classification as a highly potent and selective MOR agonist which is strongly supported by in vitro binding [25, 26, 28, 31] and functional studies [13, 14, 24–26, 28], antagonism of its in vitro [14, 23, 26] and in vivo effects by naloxone, naltrexone and selective MOR antagonists [14, 25, 31, 34], and antisense mapping studies [31], it is evident that 14-methoxymetopon has pharmacological and functional profiles distinct from those of traditional MOR agonists.

 The effect of the replacement of the 5-methyl group in 14-methoxymetopon (**13**) by a benzyl group resulting in analogue **15** was examined aiming for understanding the role of the substitution pattern in position 5 in *N*-methylmorphinan-6-ones on the interaction with opioid receptors [14]. Exchanging the 5-methyl group in **13** with a benzyl group in **15**, left binding affinities at DOR and KOR largely unaffected, while retaining the high affinity at the MOR (K_i_ of 0.15 nM for **13** versus 0.31 nM for **15**), and the MOR selectivity (Table 1) [14]. On the basis of in vitro [^35^S]GTP*γ*S functional findings, the presence of a 5-benzyl group in compound **15** yielded a new MOR agonist, showing a five times increase in potency compared to its 5-methyl substituted derivative **13**; while it was slightly less efficacious in stimulating [^35^S]GTP*γ*S binding, but displaying similar efficacy to morphine (Table 2) [14]. The binding affinity of analogue **15** for opioid receptors is comparable to that of 14-methoxymetopon **13**; it is hence likely that the presence of an arylalkyl group such as benzyl at position 5 leads to an increase in agonist potency. A similar profile was noted when comparing in vitro agonist properties of the 5-benzyl analogue **15** to its 5-unsubstituted derivative 14-*O*-methyloxymorphone (**1**) (Tables 1 and 2). Compound **15** produced effective and naloxone-sensitive analgesia in the hot-plate and tail-flick tests in mice after s.c. administration [14]. Its antinociceptive potency was less than two times lower than that of 14-methoxymetopon **13**, and 50 times higher than that of morphine (Tables 1 and 2) [14]. Substitution of the 5-methyl group in **13** with a benzyl group appeared to be well tolerated leading to a highly potent and efficacious MOR antinociceptive agent. Besides, the introduction of a benzyl substituent at position 5 in 14-*O*-methyloxymorphone (**1**), giving rise to compound **15**, produced a three times decrease in antinociceptive potency (Table 2). Further experiments performed with this new opioid molecule showed that contrary to morphine, 14-*O*-methyloxymorphone (**1**) and 14-methoxymetopon (**13**), no significant alteration in motor coordination was induced by derivative **15 **in the mouse rotarod test at any of the s.c. analgesic doses (Table 2) [14]. Results from pharmacological investigations have shown that exchanging the 5-methyl with a benzyl group in 14-methoxymetopon (**13**) afforded a potent MOR antinociceptive agent with decreased propensity to cause locomotor dysfunction.

Subsequent synthetical and biological work targeted 14-arylalkyloxymetopon derivatives of 14-methoxymetopon (**13**), resulting in 14-benzyloxymetopon (**18**) [13] and 14-phenylpropoxy-substituted analogues **20** and PPOM (**21)** [22]. The benzyloxy or phenylpropoxy substitution in position 14 gave rise to new structures, **18** and **21**, respectively, which had markedly enhanced binding affinities at both DOR and KOR, while the high affinity at MORs remains unaffected compared to the parent compound **13** (Table 1). As a result, the MOR selectivity was markedly reduced for the benzyloxy derivative **18**, with a complete loss of MOR selectivity for PPOM (**21**), while some MOR selectivity was still depicted by its 3-methoxy analogue **20**. All three 5-methyl, 14-arylalkyloxy-substituted compounds, **18**, **20,** and **21**, showed high antinociceptive activity when administered s.c. to mice in pain models including hot-plate, tail-flick, and writhing tests (Table 1) [13, 22]. Notable was the observation, that in vivo, PPOM (**21**) was an extremely potent opioid agonist exhibiting substantially augmented analgesic potency compared not only to its 14-methoxy analogue **13** and morphine, but it was even more effective in inducing analgesia than etorphine (Table 4), a MOR morphinan used in veterinary medicine for anesthesia of large animals and wildlife species [36, 37]. Moreover, the 3-*O*-methyl ether of PPOM (**20**) also showed several hundredfolds greater analgesic potency than morphine, although compared to PPOM (**21**), its potency was 10 to 55 times lower depending upon the applied analgesic assay and displaying similar potency to etorphine (Table 4) [22].

Assessment of physicochemical properties has gained increased relevance in drug development, particularly in understanding the behavior of bioactive molecules and correlation with their biological profiles [38, 39]. The lipophilicity of some *N*-methylmorphinan-6-ones including 14-*O*-methyloxymorphone (**1**), 14-methoxymetopon (**13**) and its 5-benzyl analogue **15** was experimentally evaluated in comparison to morphine (Table 2) and oxymorphone [24]. While similar lipophilicity is shown by 14-*O*-methyloxymorphone (**1**) and its parent molecule oxymorphone (log *P* values of 0.60 and 0.67, resp.) and also morphine, the presence of a 5-methyl group in compound **13** leads to a more lipophilic compound (Table 2). Further increase in lipophilicity resulted after replacement of the 5-methyl with a 5-benzyl group in derivative **13 **(Table 2), making such structures feasible candidates for oral and/or transdermal delivery.

## 4. Conclusion

The summarized research reports on the development of novel opioid analgesics from the class of *N*-methylmorphinan-6-ones have highlighted the spectrum of chemical strategies, biological and pharmacological properties and ligand-based SARs, directed towards the discovery of more effective and safer pain medications. Targeting position 5 in 14-alkoxy-*N*-methylmorphinas-6-ones represents a promising approach for tuning activities and influencing interaction with opioid receptors in this class of compounds. We reported that the presence of methyl and benzyl groups at position 5 gives rise to strong opioid antinociceptive agents with reduced propensity to induce undesired effects compared to morphine although interacting selectively with MORs. The promising experimental results represent a useful and valuable aspect for design and optimization of existing structural templates increasing the chance of identifying clinically useful analgesics for superior management of pain.

## Figures and Tables

**Table 1:** In vitro and in vivo opioid activity and SAR study on the variation of the substituent in position 5 in 14-alkoxy-substituted *N*-methylmorphinan-6-ones.

		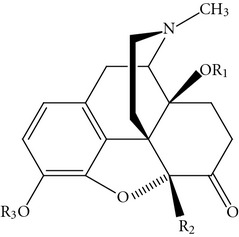								

		Binding affinity (K_i_, nM)					Agonist potency (relative to morphine)		
	R_1_, R_2_, R_3_	MOR^a^	DOR^b^	KOR^d^	Selectivity		MVD^e^		GPI^f^
					DOR/MOR	KOR/MOR			

									
Oxymorphone	H, H, H	0.97	80.5	61.6	83	63		0.4	ND
									

									
**1**	Me, H, H	0.10	4.80	10.2	48	102		331	156
									

									
**13**	Me, Me, H	0.15	13.3	25.2	89	168		202	52
									

**14**	Et, Me, H	0.46	12.2^c^	43.2	26	94		ND	49^g^

**15**	Me, Bz, H	0.31	13.1	22.8	42	73		ND	ND
									

**18**	Bz, Me, H	0.18	3.67	2.46	20	14		696	167

									
**20**	PhPr, Me, Me	0.62	6.33	25.0	10	40		ND	ND
									

									
**21**	PhPr, Me, H	0.20	0.14	0.40	0.7	2		ND	ND
									

Bz: benzyl; Et: ethyl; Me: methyl; PhPr: phenylpropyl; K_i_: inhibition constant; ND: not determined.

^a^Binding against [^3^H]DAMGO in rat brain membranes [13, 14, 22, 23, 25, 26].

^b^Binding against [^3^H][Ile^5,6^]deltorphin II or ^c^[^3^H]DSLET in rat brain membranes [13, 14, 22, 23, 25, 26].

^d^Binding against [^3^H]U69,593 in rat or guinea pig brain membranes [13, 14, 22, 23, 25, 26].

^e^Determined in the MVD [13, 23, 24].

^f^Determined in the GPI [13, 23].

^g^Relative to normorphine [25].

^h^Determined in the hot-plate test in mice after s.c. administration [12–14, 22].

^i^Determined in the tail-flick test in mice after s.c. administration [14, 22, 24].

^j^Determined in the writhing test in mice after s.c. administration [20, 22, 25].

**Table 2:** Comparison of pharmacological and physicochemical properties of morphine, 14-*O*-methyloxymorphone **(1)**, 14-methoxymetopon **(13),** and its 5-benzyl-substituted analogue **15**.

	Agonist activity^a^	Antinociception	Motor coordination^d^	Log *P* ^g^
	[^35^S]GTP*γ*S (EC_50_, nM; % stim)	ED_50_ (mg/kg)		
Morphine	462; 85	2.63^b^ 2.29^c^	10^e^	0.88

**1**	23.7; 108	0.017^b^ 0.014^c^	0.06^e^	0.60

**13**	63.0; 110	0.028^b^ 0.028^c^	0.1^e^	1.12

**15**	13.7; 85	0.053^b^ 0.043^c^	0.2^f^	1.49

EC_50_: effective concentration necessary to produce a 50% effect; ED_50_: effective analgesic dose to produce a 50% effect; log *P*: partition coefficient.

^a^Determined in [^35^S]GTP*γ*S functional assays in rat brain membranes; Data as % stimulation relative to DAMGO [14].

^b^Determined in the hot-plate test in mice after s.c. administration [14].

^c^Determined in the tail-flick test in mice after s.c. administration [14].

^d^Determined in the rotarod test in mice after s.c. administration [14].

^e^Significant decrease [14].

^f^No significant effect [14].

^g^The experimental log *P* determined in octanol/water [14].

**Table 3:** Overview of the analgesic activity of 14-methoxymetopon **(13)** in various models of pain in rodents.

Pain model	Route	ED_50_	Potency versus morphine	Reference
Hot-plate test (mouse)				
50°C	s.c.	54 *μ*g/kg	24	[29]
52°C	s.c.	28 *μ*g/kg	94	[14]
56°C	s.c.	30 *μ*g/kg	28	[30]

Hot-plate test 55°C (rat)	s.c.	15 *μ*g/kg	313	[25]

	s.c.	30 *μ*g/kg	63	[30]
	s.c.	28 *μ*g/kg	82	[14]
Tail-flick test (mouse)	s.c.	7.6 *μ*g/kg	500	[31]
	i.c.v.	0.29 fg/animal	>1,000,000	[31]
	i.t.	0.31 fg/animal	>1,000,000	[31]

Tail-flick test (rat)	s.c.	7.2 *μ*g/kg	250	[25]
	i.p.	40 *μ*g/kg	125	[25]

Tail electric stimulation test (rat)	s.c.	30 *μ*g/kg^a^	167	[33]

Writhing test (mouse)				
acetic acid	s.c.	7 *μ*g/kg	99	[25]
PPQ	s.c.	9 *μ*g/kg	44	[30]

Carrageenan-induced inflammatory pain (rat)	s.c.	20 *μ*g/kg^a^	100	[35]

^a^Significant effect.

**Table 4:** Comparison of antinociceptive potencies of 14-methoxymetopon (**13**) and its 14-phenylpropoxy substituted analogues **20** and **21**, with morphine and etorphine.

	ED_50_ (*μ*g/kg)^a^		
	Hot-plate test	Tail-flick test	PPQ writhing test

**20**	2.6	4.4	1.7
**21**	0.10	0.08	0.16
**13**	30	30	9.0
Morphine	850	1,920	400
Etorphine	1.0	2.0	0.40

^a^Determined in mice after s.c. administration [22].

## Abbreviations

[^35^S]GTP*γ*S: Guanosine-5′-O-(3-[^35^S]thio)-triphosphate
DMF: *N,N*-dimethylformamide
DOR: *δ* opioid receptor
EC_50_, IC_50_: Concentration necessary to produce a 50% effect
ED_50_: Effective analgesic dose necessary to elicit a 50% effect
GPI: Guinea pig ileum
i.c.v.: Intracerebroventricular
i.p.: Intraperitoneal
i.t.: Intrathecal
i.v.: Intravenous
K_i_: Inhibition constant
KOR: *κ* opioid receptor
MOR: *μ* opioid receptor
MVD: Mouse vas deferens
NaH: Sodium hydride
PPOM: 14-Phenylpropoxymetopon
PPQ: Paraphenylquinone
RVD: Rat vas deferens
SAR: Structure-activity relationship
s.c.: Subcutaneous.
